# Dynorphin and GABA_A_ Receptor Signaling Contribute to Progesterone’s Inhibition of the LH Surge in Female Mice

**DOI:** 10.1210/endocr/bqaa036

**Published:** 2020-03-17

**Authors:** Yali Liu, Xiaofeng Li, Xi Shen, Deyana Ivanova, Geffen Lass, Wen He, Qiuju Chen, Sha Yu, Yun Wang, Hui Long, Li Wang, Qifeng Lyu, Yanping Kuang, Kevin T O’Byrne

**Affiliations:** 1 Department of Assisted Reproduction, Shanghai Ninth People’s Hospital Affiliated to Shanghai Jiaotong University School of Medicine, Huangpu District, Shanghai, China; 2 Department of Women and Children’s Health, Faculty of Life Sciences and Medicine, King’s College London, Guy’s Campus, UK; 3 Shanghai First Maternity and Infant Hospital, Tongji University School of Medicine, Pudong New Area, Shanghai, China

**Keywords:** progesterone, LH surge, AVPV, dynorphin, GABA

## Abstract

Progesterone can block estrogen-induced luteinising hormone (LH) surge secretion and can be used clinically to prevent premature LH surges. The blocking effect of progesterone on the LH surge is mediated through its receptor in the anteroventral periventricular nucleus (AVPV) of the hypothalamus. However, the underlying mechanisms are unclear. The preovulatory LH surge induced by estrogen is preceded by a significant reduction in hypothalamic dynorphin and gamma-aminobutyric acid (GABA) release. To test the detailed roles of dynorphin and GABA in an LH surge blockade by progesterone, ovariectomized and 17β-estradiol capsule-implanted (OVX/E_2_) mice received simultaneous injections of estradiol benzoate (EB) and progesterone (P) or vehicle for 2 consecutive days. The LH level was monitored from 2:30 pm to 8:30 pm at 30-minute intervals. Progesterone coadministration resulted in the LH surge blockade. A continuous microinfusion of the dynorphin receptor antagonist nor-BNI or GABA_A_ receptor antagonist bicuculline into the AVPV from 3:00 pm to 7:00 pm reversed the progesterone-mediated blockade of the LH surge in 7 of 9 and 6 of 10 mice, respectively. In addition, these LH surges started much earlier than the surge induced by estrogen alone. However, 5 of 7 progesterone-treated mice did not show LH surge secretion after microinfusion with the GABA_B_ receptor antagonist CGP-35348. Additionally, peripheral administration of kisspeptin-54 promotes LH surge-like release in progesterone treated mice. These results demonstrated that the progesterone-mediated suppression of the LH surge is mediated by an increase in dynorphin and GABA_A_ receptor signaling acting though kisspeptin neurons in the AVPV of the hypothalamus in female mice.

Reproductive cyclicity in mammals is regulated by the interplay between estradiol (E_2_), progesterone (P), gonadotropin-releasing hormone (GnRH), and gonadotropin hormones (1). Progesterone is produced by the corpus luteum and inhibits hypothalamic GnRH and consequently gonadotropin secretion (2). Moreover, when administered before or concurrent with E_2_, P inhibits E_2_ positive feedback and abolishes the preovulatory GnRH/luteinising hormone (LH) surge (1, 3). This inhibition of the LH surge by P has been identified in many species, including rodents (3), sheep (4), primates (5), and humans (6–8). Clinically, this P effect has been used as an alternative to a gonadotropin-releasing hormone (GnRH) analogue for suppressing premature LH surges in controlled ovarian stimulation (COS) for in vitro fertilization (IVF) cycles (6–8). Despite its physiological and clinical importance, the mechanism by which P blocks the preovulatory LH surge is not fully understood.

Progesterone has been shown to block the LH surge by acting centrally to inhibit the surge of GnRH secreted by the hypothalamus (4). In rodents, the hypothalamic nuclei that control positive and negative feedback are different. The anteroventral periventricular nucleus (AVPV) is thought to underlie the LH surge by mediating the positive feedback effects of E_2_ and the arcuate nucleus (ARC), which contains kisspeptin/neurokinin-B/dynorphin (KNDy) neurons and mediates steroid negative feedback actions on pulsatile LH secretion (9, 10). A recent study showed that progesterone’s inhibitory effects on the LH surge are mediated by its receptor in the AVPV (11). The lack of colocalization of P receptors (PRs) with GnRH neurons (12) suggests that this action must occur via other neurons expressing steroid receptors. The control of GnRH activity and subsequent LH release involves numerous neurotransmitter systems (13), including the dynorphin (14) and gamma-aminobutyric acid (GABA) (15) pathways, which modulate GnRH neurons within the hypothalamus.

It is known that the majority of dynorphin neurons express PR messenger ribonucleic acid (mRNA) (16). It is generally thought that P inhibits GnRH pulse frequency though dynorphin neurons (17, 18). However, the role that dynorphin plays on LH surge regulation is controversial. Previous studies have suggested that a decrease in dynorphin inhibitory input to the preoptic area (POA) is a prerequisite for LH surge secretion in rodents (19), and by acting through kappa-opioid receptors (KOR), dynorphin could block the LH surge and ovulation (20). However, another study has demonstrated that a substantial k-opioid tone is still present during LH surge initiation (21).

Additionally, GABA plays an important role before the occurrence of the LH surge (22). Gamma-aminobutyric acid neurons in the paraventricular nucleus and POA express PRs in monkeys (23). The infusion of a GABA agonist into the POA in rats has been shown to attenuate the LH surge (24). It is noteworthy that GABA can mediate both excitation and inhibition through the ionotropic receptor GABA_A_, depending on the intracellular chloride concentration of the GnRH neurons and inhibition through the metabotropic receptor GABA_B_ (25, 26). However, whether GABA, especially GABA located in the AVPV, is involved in P inhibition of the LH surge is still unclear.

In the present study, we monitored LH surge profiles in OVX/E_2_ capsule + estradiol benzoate (EB) + P-treated female mice subjected to a bilateral continuous microinfusion of a potent KOR (nor-BNI), GABA_A_ receptor (bicuculline [BIC]), or GABA_B_ receptor (CGP-35348) antagonist, respectively, into the AVPV, to investigate the potential rescue of the P-induced inhibition of the LH surge by the decreased activity of the dynorphin and GABA signaling pathways in the AVPV. Additionally, we monitored LH secretion in OVX/E_2_ capsule + EB + P-treated female mice receiving peripheral administration of kisspeptin-54 to investigate whether the ability of GnRH neurons to respond to kisspeptin remains intact.

## Materials and Methods

### Animals

Female, adult (10 weeks old) C57BL/6 mice weighing 20–25 g were obtained from Charles River (Margate, UK), housed individually under controlled temperature (22 ± 2 °C) and light conditions (12:12 hours light:dark, with lights on at 7:00 am), and given a standard maintenance diet (Special Dietary Services, Wittam, UK) and water ad libitum. All procedures were conducted in accordance with the British Home Office Animals Scientific Procedures Animals Act 1986, and all experimental protocols were approved by the Animal Welfare and Ethical Review Body at King’s College London.

### Surgical procedures

All surgical procedures were carried out under anaesthesia with ketamine (100 mg/kg intraperitoneal [i.p.]; Pharmacia and Upjohn Ltd., Crawley, UK) and Rompun (10 mg/kg i.p.; Bayer, Leverkusen, Germany). Only mice that had exhibited at least 3 consecutive 4- to 5-day estrous cycles were selected for experimentation. They were subjected to bilateral OVX and implanted with a silastic capsule (Sanitech, Havant, UK) containing 17β-estradiol (1 μg per 20 g of body weight) (27). To assess the effects of dynorphin and GABA antagonists on the hypothalamic AVPV, animals were secured in a motorized Kopf stereotaxic frame and surgical procedures were performed using a robotic stereotaxic system (Neurostar, Tubingen, Germany). A small hole was drilled in the skull at a location above the AVPV. The stereotaxic coordinates used to target the AVPV (0.25 mm lateral, 0.26 mm anterior to the bregma and at a depth of 5.3 mm below the surface of the skull) were obtained from the Paxinos and Franklin mouse brain atlas (28). A bilateral guide cannula (26-gauge; Plastics One, Roanoke, Virginia) was then targeted towards the AVPV at the same time as the OVX surgery. The guide cannula was secured using dental cement (Dental Filling Ltd., Swindon, UK) and fitted with a dummy cannula (Plastics One) to maintain patency. The mice were housed in individually ventilated cages after surgery and other parameters were controlled in each cage.

### Blood sampling procedure

After a 3- to 5-day recovery period postsurgery, the mice were handled daily to acclimatize them to the tail-tip blood sampling procedure (29). The tip of the mouse’s tail was excised using a sterile scalpel for subsequent blood sample collection (30). After a 1-hour acclimation period, blood samples (4 µl) were collected at 30 minute intervals using a pipette tip dipped in heparinized saline (50 U/ml heparin sodium/ml normal saline; CP Pharmaceuticals, Wrexham, UK), and the whole blood was immediately diluted with 56 μl of 0.2% BSA in 1 M PBS containing 0.05% Tween 20 (PBS-T), vortexed, and snap frozen on dry ice. Blood samples were frozen at -80 °C for a later assay to determine LH concentrations.

### Effect of an intra-AVPV infusion of nor-BNI, BIC, or CGP-35348 on progesterone-mediated inhibition of the LH surge

**Figure 1.**
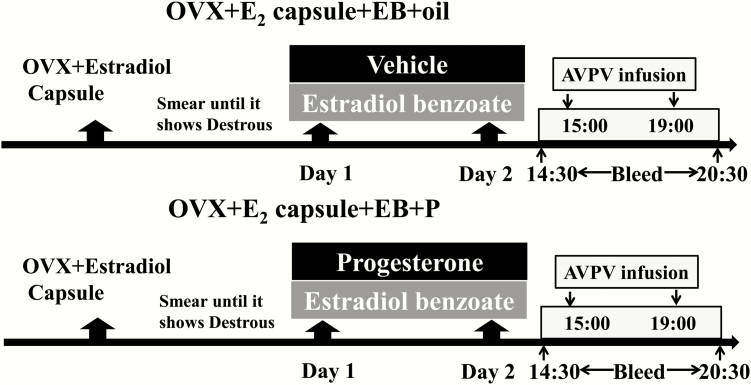
**Timeline of the experiments.** Ovariectomized mice implanted with estradiol (E_2_) capsules (OVX/E_2_) and showing a diestrous-like vaginal cytology were administered a sc injection of estradiol benzoate (EB: 1 μg/20 g of body weight) and an i.p. injection of progesterone (P: 100 μg/20 g of body weight) at 8:30 am, followed by a second i.p. injection of P at 4:30 pm (Day 1). The following day, designated Day 2, this hormone regime was repeated. As positive controls, the OVX/E_2_+EB-treated mice were injected with vehicle (50 μl of peanut oil, i.p.,) instead of P. Mice were administered an intra-AVPV infusion of relevant drugs or artificial cerebrospinal fluid as control from 3:00 pm to 7:00 pm on Day 2. Blood samples were collected every 30 minutes between 2:30 pm and 8:30 pm on Day 2 from both experimental groups and assayed for LH.

The treatment of OVX/E_2_ capsule-implanted mice with EB has been validated to induce an LH surge (30) and facilitates the study of the mechanisms underlying this key reproductive process. Six to 10 days after the OVX/E_2_ surgery, mice with a diestrous-like vaginal cytology were administered a subcutaneous (sc) injection of EB (1 μg/20 g of body weight) and an i.p. injection of P (100 μg/20 g of body weight) at 8:30 am, followed by a second i.p. injection of P at 4:30 pm. This is designated Day 1 of treatment. The following day, Day 2, this hormone regime was repeated, and is an established protocol that results in the inhibitory effect of P on the estrogen-induced LH surge (3, 11). The dose of P was chosen to maintain a serum P level higher than the level after ovulation (11, 31). As positive controls for the inhibitory effects of P, a separate group of OVX/E_2_ capsule + EB-treated mice were injected with vehicle (50 μl of peanut oil, i.p., Sigma-Aldrich, Gillingham, UK) instead of P. These steroid regimes are illustrated in Fig. 1.

Intra-AVPV injections of a selective antagonist for the dynorphin activated KOR (nor-BNI; Tocris Bioscience, Bristol, UK), GABA_A_ receptor antagonist (BIC; Sigma-Aldrich), GABA_B_ receptor antagonist (CGP-35348; Sigma-Aldrich), or artificial cerebrospinal fluid (aCSF) were administered to separate groups of OVX/E_2_ capsule + EB + P mice to identify the roles of dynorphin and GABA signaling in the P-mediated inhibition of the LH surge. On the afternoon of Day 2 (Day 1 is the day on which the EB and P treatments started), a bilateral internal cannula (Plastics One) with extension tubing preloaded with nor-BNI, BIC, CGP-35348, or aCSF was inserted into the guide cannula and extended 0.5 mm beyond the guide cannula tip to reach the AVPV. The distal ends of the tubing were extended outside of the animal cage and connected to 10-μl Hamilton syringes (Waters Ltd., Elstress, UK) secured in a PHD 2000 programmable syringe pump (Harvard Apparatus, Massachusetts), thereby allowing constant infusion without disturbing the animals during the experiment.

The infusion dose for nor-BNI (0.1 μg administered at a rate of 300 nl/h in aCSF on each side) (32), BIC (6 ng administered at a rate of 300 nl/h in aCSF on each side) (33) or CGP-35348 (0.4 µg administered at a rate of 300 nl/h in aCSF on each side) (34) was based on previous research and our preliminary studies. The infusion was performed from 3:00 pm to 7:00 pm on Day 2. The lights-on phase in our laboratory is from 7:00 am to 7:00 pm, and as 2 hours after the midpoint of the light phase corresponds to the beginning of the critical period for ovulation block by pentobarbital (35), and significant LH surge levels and cfos levels in GnRH neurons and the AVPV is around the light/dark transfer period (36), we chose 3:00 pm to 7:00 pm as the treatment infusion period in our experiments.

The OVX/E_2_+EB + P-treated mice were administered nor-BNI (n = 12), BIC (n = 12), CGP-35348 (n = 10) or aCSF (n = 12) via the intra-AVPV cannulae from 3:00 pm to 7:00 pm and blood samples were collected every 30 minutes between 2:30 pm and 8:30 pm on Day 2, the expected day of the LH surge (Day 1 is the day on which the EB and P treatments began). In addition, a separate group of OVX/E_2_+EB+oil (positive control; n = 12) were administered an intra-AVPV infusion of aCSF from 3:00 pm to 7:00 pm (300 nl/h of aCSF on each side) on Day 2 as controls. All of the mice in each experiment were independent and used only once. Blood samples were collected every 30 minutes between 2:30 pm and 8:30 pm on Day 2 (Fig. 1).

### Effect of peripheral administration of kisspeptin-54 on progesterone-mediated inhibition of the LH surge

Separate groups of OVX/E_2_ capsule + EB + P-treated mice received a single i.p. injection of Kisspeptin-54 (Kp-54, Tocris Bioscience; 1 nmol / 30 g of body weight in 100 μl) (37) (n = 9), or vehicle saline (n = 5) at 3:30 pm on Day 2 of the protocol as shown in Fig. 1. Blood samples were collected every 30 minutes between 2:30 pm and 8:30 pm on Day 2 as described above.

### Brain collection and histological verification of the cannula position

After experimentation, 0.5 µl of India ink was injected over 5 minutes through the internal cannulae inserted into the guide cannulae for the purpose of site verification. Animals were then euthanized by decapitation. The brain was removed, snap frozen on dry ice, and then stored at -80 °C, followed by sectioning (30 µm) using a cryostat (Bright Instrument Co., Ltd., Luton, UK). Every third section throughout the AVPV region corresponding to the bregma (0.74 to -0.7 mm) was mounted and stained with cresyl violet to evaluate the cannula position. The slides were then viewed under a light microscope and the images were captured using a digital camera (Zeiss, Oberkochen, Germany). Only data from animals with correct cannula placement were analyzed. The remaining mice were excluded from the analysis due to inaccurate probe placement.

### LH measurement

Blood samples were assessed using an enzyme-linked immunosorbent assay (ELISA), as previously reported (29). A mouse LH standard (mLH; reference preparation, AFP-5306A, NIDDK-NHPP, Bethesda, Maryland), coating antibody (https://antibodyregistry.org/RRID:AB_2665514, monoclonal antibovine LH beta subunit antiserum, 518B7, University of California, California) (38), anti-LH antibody (https://antibodyregistry.org/RRID:AB_2665533; National Hormone & Peptide Program, Torrance, California) (39), and a secondary antibody (http://antibodyregistry.org/RRID:AB_772206, GE Healthcare, Chicago, Illinois) (40) were used to determine the LH concentration. The intra-assay and inter-assay variations were 4.6% and 10.2%, respectively.

### Statistical analysis

Statistical comparisons of the separate LH values from 2:30 pm to 8:30 pm between groups were performed using one-way ANOVA followed by posthoc Tukey’s multiple comparison test or the Games–Howell test for data with unequal variance. Data are presented as the means ± SEM, and *P* < 0.05 was considered statistically significant.

## Results

### Cannula placement in the AVPV

**Figure 2.**
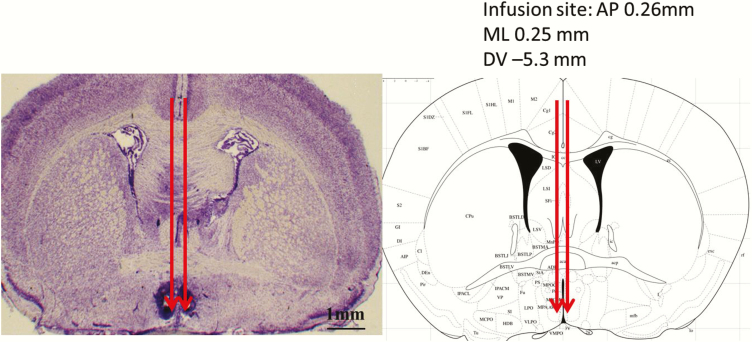
**Photomicrograph of the cannula-targeted sites in the hypothalamic anteroventral periventricular nucleus (AVPV)**. Photomicrograph of a cresyl violet-stained coronal section showing representative examples of bilateral cannulae placement in the AVPV. The arrows indicate the site of the cannulae.

The location of each intra-AVPV cannula was confirmed by microscopic histological inspection of cresyl violet-stained brain sections. Only animals with appropriate bilateral cannula placement in the AVPV were included in the analysis (Fig. 2). Of the 58 mice that underwent hypothalamic cannulation, 45 were confirmed to have correct bilateral cannula placement in the AVPV. Data from animals with incorrect cannula placement, either on one or both sides were excluded from the analysis. These included: 3 of the 12 OVX/E_2_+EB + P-treated administered nor-BNI; 2 of 12 OVX/E_2_+EB + P-treated administered BIC; 3 of 10 OVX/E_2_+EB + P-treated administered CGP-35348; 3 of 12 OVX/E_2_+EB + P-treated administered aCSF; and 2 of 12 OVX/E_2_+EB+oil-treated administered aCSF.

### Effects of progesterone on the LH surge in OVX/E_2_+EB-treated mice

**Figure 3.**
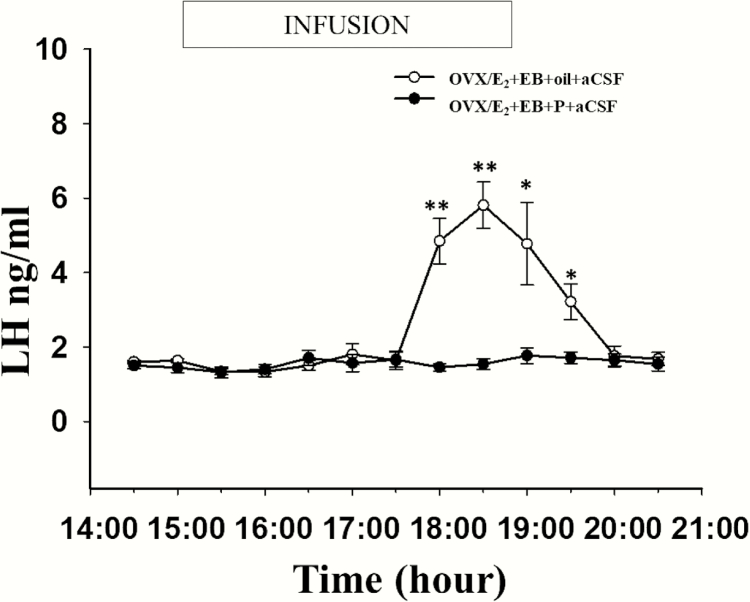
**The inhibitory effects of progesterone (P) on the LH surge in OVX/E**_**2**_  **capsule + EB mice.** Eight of the 10 OVX/E_2_+EB+oil-treated mice receiving intra-AVPV infusion of artificial cerebrospinal fluid (aCSF) between 3:00 pm and 7:00 pm on designated Day 2 (see Fig. 1 for steroid hormone protocol) and showed the expected LH surge approximately 1 hour before lights off (7:00 pm). The remaining 2 animals failed to show an LH surge (data not shown). All 9 of the OVX/E_2_+EB group treated with P (see Fig. 1) and receiving intra-AVPV infusion of aCSF on Day 2 failed to show an LH surge (same data illustrated in Fig. 3). Mean ± SEM levels of LH are indicated. Significantly different values are indicated by an asterisk (*P < 0.05; **P < 0.01).

Ten mice with correct intra-AVPV cannula placement underwent the OVX/E_2_+EB+oil surge induction protocol and aCSF infusion with tail-tip blood sampling every 30 minutes for 6 hours (from 2:30 pm to 8:30 pm). Of these animals, 8 exhibited an LH surge (Fig. 3) beginning at approximately 5:30 pm before light offs (7:00 pm). Luteinising hormone concentrations prior to surge onset were 1.52 ± 0.05 ng/ml and gradually increased to peak concentrations of 5.81 ± 0.62ng/ml just before (0.5 hour) lights out, and then was gradually reduced to baseline levels 1.5 hours later. The remaining 2 mice showed no change in LH secretion during the blood sampling period (data not shown). However, P successfully blocked the LH surge, in all 9 OVX/E_2_+EB-treated mice with correct intra-AVPV cannula placement that were administered P together with intra-AVPV aCSF infusion (Fig. 3).

### Effect of an intra-AVPV infusion of nor-BNI on the LH surge profile in OVX/E_2_+EB + P-treated mice

**Figure 4.**
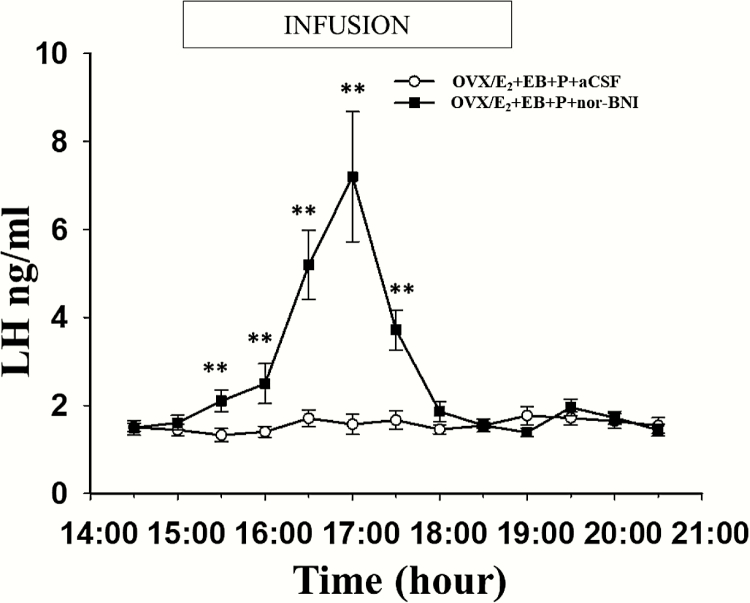
**Microinfusion of the kappa opioid receptor antagonists, nor-BNI into the AVPV reversed the inhibitory effects of progesterone (P) on the LH surge in OVX/E**_**2**_  **capsule + EB treated mice**. Mean (±SEM) LH levels in 7 of 9 OVX/E_2_ capsule + EB + P-treated mice receiving a bilateral intra-AVPV infusion of nor-BNI (0.1 μg administered at a rate of 300 nl/h in artificial cerebrospinal fluid [aCSF] on each side) between 3:00 pm and 7:00 pm on designated Day 2 (see Fig. 1 for experimental timeline) reveal a typical LH surge, but advanced in onset by approximately 2.5 hours. The remaining 2 animals failed to show an LH surge (data not shown). All 9 of the OVX/E_2_+EB group treated with P (see Fig. 1) and receiving intra-AVPV infusion of aCSF on Day 2 failed to show an LH surge (same data illustrated in Fig. 3). Significantly different values are indicated by an asterisk (*P < 0.05; **P < 0.01).

The intra-AVPV infusion of nor-BNI rescued the LH surge in 7 of 9 mice with correct intra-AVPV cannula placement that underwent the OVX/E_2_+EB + P protocol (Fig. 4). A significant increase in mean LH concentration was observed between 3:30 pm and 5:30 pm in the EB + P-treated plus nor-BNI infusion group than in the mice subjected to the same protocol but infused with aCSF as control (Fig. 4). The remaining 2 mice showed no change in LH secretion during the blood sampling period (data not shown). In the nor-BNI infusion group, which showed an LH surge, the mean LH peak levels have no significant difference with those of OVX/E_2_+EB-treated with aCSF infusion mice (7.2 ± 1.48 vs. 5.81 ± 0.62 ng/ml, respectively, *P* > 0.05). However, the nor-BNI infusion group exhibited LH surges much earlier than the OVX/E_2_+EB-treated group, with the peak of the LH surge in the nor-BNI group occurring approximately 1.5 hours earlier (compare Figs. 3 and 4).

### Effect of an intra-AVPV infusion of bicuculline or CGP-35348 on the LH surge in OVX/E_2_+EB + P-treated female mice

**Figure 5.**
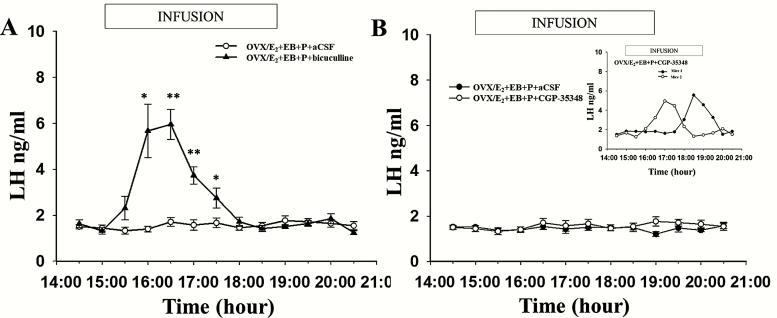
**Microinfusion of GABA**_**A**_  **antagonist, bicuculline, or GABA**_**B**_  **antagonist, CGP-35348 into the AVPV in OVX/E**_**2**_  **capsule + EB + P-treated mice**. **A:** Mean (±SEM) LH levels in 6 of 10 OVX/E_2_ capsule + EB + P-treated mice receiving a bilateral intra-AVPV infusion of bicuculline (6 ng administered at a rate of 300 nl/h in aCSF on each side) between 3:00 pm and 7:00 pm on designated Day 2 (see Fig. 1 for experimental timeline) reveal a typical LH surge, but advanced in onset by approximately 2.5 hours. The remaining 4 animals failed to show an LH surge (data not shown). All 9 of the OVX/E_2_ capsule + EB + P-treated group receiving intra-AVPV infusion of aCSF as control failed to show an LH surge (same data illustrated in Fig. 3). **B:** Mean (±SEM) LH levels in 5 of the 7 OVX/E_2_ capsule + EB + P-treated mice receiving a bilateral intra-AVPV infusion of CGP-35348 (0.4 µg administered at a rate of 300 nl/h in aCSF on each side) between 3:00 pm and 7:00 pm on designated Day 2 (see Fig. 1 for experimental timeline) failed to show an LH surge, as did the aCSF treated controls (same data illustrated in Figs. 3 and 5A). The remaining two OVX/E_2_ capsule + EB + P-treated mice receiving intra-AVPV infusion CGP-35348 showed LH surges (B insert). Significantly different values are indicated by an asterisk (*P < 0.05; **P < 0.01).

An intra-AVPV infusion of bicuculline rescued the surge in 6 of 10 OVX/E_2_+EB + P-treated mice with correct intra-AVPV cannula placement (Fig. 5A). Moreover, the surge began approximately 2 hours earlier, starting at about 3:00 pm and returning to baseline at 6:00 pm, compared to the surge in the OVX/E_2_+EB+oil-treated group administered intra-AVPV aCSF (compare Fig. 5A with Fig. 3). The peak LH surge levels in bicuculline infusion groups were similar to the mice in the OVX/E_2_+EB with aCSF infusion group (5.95 ± 0.66 vs. 5.81 ± 0.62 ng/ml, respectively, *P* > 0.05). The remaining 4 mice showed no change in LH secretion during the blood sampling period (data not shown). Of the 7 OVX/E_2_+EB + P-treated mice with correct intra-AVPV cannula placement that underwent intra-AVPV infusion of CGP-35348, 5 did not show an LH surge during the blood sampling period from 2:30 pm to 8:30 pm (Fig. 5B). Only 2 mice exhibited an LH surge: 1 showed an LH surge starting at approximately 3:30 pm, reaching a peak value of 4.95ng/nl at 5:00 pm and then gradually returning to baseline at 6:30 pm, while the other one exhibited an LH surge at 5:30 pm, that is, at a similar time to the OVX/E_2 _+ EB with aCSF infusion, and reached a peak value of 5.56 ng/ml 1h later that finally ended at 8:00 pm (Fig. 5B).

### Effect of peripheral administration of kisspeptin-54 on progesterone-mediated inhibition of the LH surge

**Figure 6.**
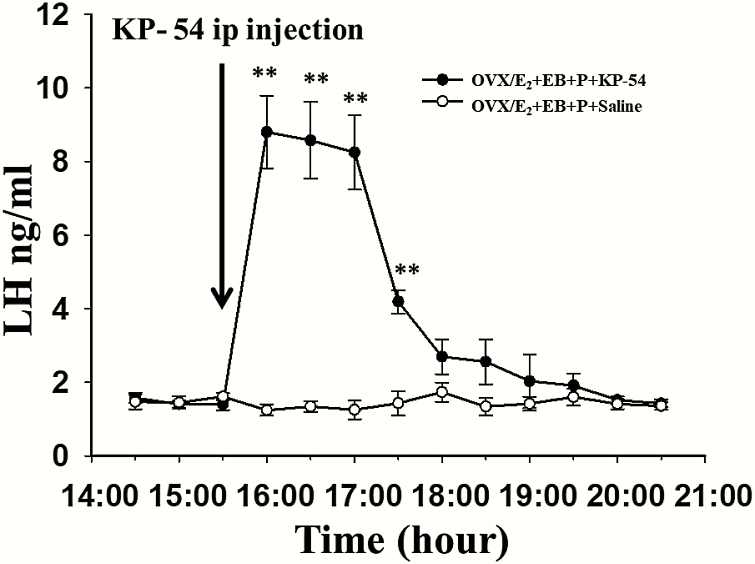
**LH response to intraperitoneal injection of kisspeptin-54 (KP-54) in OVX/E**_**2**_  **capsule + EB + P-treated mice. A:** Mean (±SEM) LH levels in the OVX/E_2_ capsule + EB + P-treated mice receiving i.p. injection of KP-54 (1 nmol / 30 g of body weight in 100 μl; n = 9) at 3:30 pm on designated Day 2 (see Fig. 1 for experimental timeline). The OVX/E_2_ capsule + EB + P-treated group (n = 5) receiving i.p. injection of saline as control failed to show increased LH secretion. Significantly different values are indicated by an asterisk (**P < 0.01).

We assessed LH secretion after of a single i.p. injection of KP-54 (1 nmol / 30 g of body weight in 100 μl; n = 9) or saline (n = 5) in the OVX/E_2_+EB + P-treated mice. KP-54 increased plasma LH levels above vehicle control immediately after injection with the highest values occuring 30 minutes postinjection (8.8 ± 0.99 vs. 1.24 ± 0.15 ng/ml, *P* < 0.001) and remaining elevated for approximateloy 2 hours postinjection (4.19 ± 0.32 vs 1.43 ± 0.33 ng/ml, *P* < 0.001) before returning to baseline (Fig. 6).

## Discussion

The results from the present study provide novel evidence that P-mediated suppression of the LH surge in female mice is mediated by dynorphin-KOR and GABA_A_ receptor signaling acting through kisspeptin neurons in the AVPV of the hypothalamus. Intra-AVPV administration of dynorphin-related KOR and GABA_A_ receptor antagonists, namely, nor-BNI and bicuculline, respectively, attenuated P-mediated inhibition of the LH surge in female mice. In contrast, GABA_B_ receptor activity in the AVPV did not seem to be closely related to P-mediated suppression of the LH surge, as microinfusion of a GABA_B_ receptor antagonist, CGP-35348, did not sufficiently rescue the P-induced inhibition of the LH surge. Moreover, peripherally administrated KP-54 induced LH surge-like secretion in OVX/E_2_+EB + P-treated mice.

Estrogen provides the signal to the GnRH/LH neurosecretory system to stimulate the LH surge in female mammals, while P can modify this response and sometimes has opposing effects (3, 41, 42). Previous studies have shown that P can block the LH surge if it is administered either coincident with or immediately after the stimulatory estradiol signal (11, 41). In accordance with these data, we confirmed that simultaneously administering a luteal phase level of P and a supraphysiological levels of EB in OVX/E_2_ mice blocks the LH surge regardless of the high estrogenic milieu by EB, which normally induces the LH surge. Studies in ewes showed that P could block the positive feedback effects by affecting the synthesis and/or secretion of the neurotransmitter systems that are targeted by E_2_ to induce the LH surge during either the activation or early transmission stages of the surge-induction process (41, 43).

The early process of LH surge production involves a considerable number of neurons and neurotransmitters. Dynorphin, as a major inhibitory neuronal system, contributes significantly to LH surge secretion in rodents (19–21). Although the majority of dynorphin-containing neurons in the AVPV coexpress both ERs and PRs (16), it is still unclear how they are regulated by steroid hormones to affect the LH surge. Reduced dynorphin levels are a prerequisite for LH surge secretion (19–21) and operate through an estrogen response element (ERE)-dependent pathway, similar to stimulation of kisspeptin expression by E_2_ in the AVPV (44). Studies have also demonstrated that significant dynorphin tone is still present during the hours when the LH surge is initiated (21), as microinjections of a KOR antagonist into the AVPV significantly increase the LH surge amplitude in estrogen-treated rats (32). Progesterone directly increases dynorphin A concentrations in the cerebrospinal fluid in sheep (45). Lustig’s study explored the role of endogenous opioid peptides (EOPs) on the inhibitory effects of P on the signal for the LH surge induced by E_2_ (46), a process that could be restored by continuous naloxone infusion (47). However, they are not site- and receptor-specific, as EOPs contain mu, delta, and kappa opioid receptors (48). The present study shows for the first time evidence for the site-specific role of dynorphin in the AVPV in mediating the inhibitory effect of P on LH surges based on the observation that the administration of the KOR antagonist, nor-BNI, directly into the AVPV rescued the LH surge in 7 of 9 mice treated with P. Moreover, compared with the positive control group, the timing of the LH surge in the nor-BNI infusion group was significantly advanced, which is consist with a previous study showing that dynorphin plays a key role in LH surge timing (19). Few GnRH neurons express KOR receptors, suggesting that dynorphin acts via other intermediates to exert its effects (49).

GABAergic neurons within the GnRH network provide an important regulatory influence on GnRH neurons (50). According to a previous study, GABA release shows a sharp decline during the preovulatory afternoon period, which is closely associated with the onset of the LH surge in female rats (51). Based on our data, GABA_A_ receptor activity in the AVPV appears to play a critical role in mediating the inhibitory effect of P on the LH surge, as intra-AVPV infusion of bicuculline, a GABA_A_ receptor antagonist, blocked the P-mediated inhibition of the LH surge in mice. There are a few possible mechanisms to explain the above data: First, P may inhibit LH secretion though allopregnanolone (52), a P metabolite that acts on GABA_A_ receptors as an allosteric agonist (52, 53). This hypothesis is supported by evidence showing that the inhibition of LH release by allopregnanolone is reversed by bicuculline in steroid-treated rats (54). Second, P may also change GABA_A_ receptor conformation and affect its synthesis and expression in a specific manner (55). Furthermore, the LH surge started much earlier in the bicuculline infusion group than in the positive control group, consistent with the preliminary conclusion that GABA_A_ receptors affect the initiation of the LH surge (56). The effect of GABA_A_ receptor activation on GnRH neurons is controversial, as a recent study showed that directly stimulating GABA neurons in the AVPV evoked a sustained large increase in LH (15). Nevertheless, infusing a GABA agonist into the POA of rats at the estimated time of the surge can effectively suppress the LH surge (24,33). GABA_A_ receptor activation has different effects on GnRH neurons in different environments, brain regions, or at different hormone levels (50, 57). Our research supports the notion that GABA_A_ receptors plays an inhibitory role in GnRH/LH secretion and for the first time provides evidence that P increases GABA levels in the AVPV to extinguish the LH surge. However, CGP-35348, a GABA_B_ receptor antagonist, did not fully rescue the LH surge, which was consistent with previous studies showing the predominance of GABA_A_ over GABA_B_ receptors in mediating LH secretion (58). The explanation for why 2 mice in the CGP-35348 treatment group produced LH surge is unclear. Perhaps this may be due to the dose of GABA_B_ receptor antagonist used, so further work is required.

Numerous neurotransmitters and neuropeptide systems co-regulate LH surge secretion. Among these, kisspeptin neurons in the AVPV nucleus plays a vital role in the regulation of the preovulatory LH surge (15). Herbison and colleagues have shown cfos labeling, a marker of neuronal activation, in both AVPV kisspeptin cells and GnRH cells at the time of the presumptive GnRH surge (59). It seems that P prevents the LH surge though interrupting estrogen-kisspeptin-GnRH signaling in the AVPV, as a previous study showed that microinjection of a P receptor antagonist into the AVPV rescued the LH surge blocked by P (11). Kisspeptin signaling is requisite and likely to be downstream of GABA_A_ receptor and dynorphin signaling, since kisspeptin neurons in AVPV which directly and potently stimulate GnRH neurons express KOR and receive direct functional GABA_A_ receptor inputs (15, 59–61). GABA generally hyperpolarizes AVPV kisspeptin neurons and there is an estradiol-induced decrease in GABAergic input to these neurons in the afternoon in ovariectomized estradiol-treated mice, allowing for the increase in kisspeptin output critical for eliciting the preovulatory LH surge (62). Leon et al (60) provided conclusive evidence that the GABA_A_ receptor blockade-induced increase in LH secretion is completely dependent on kisspeptin signaling to GnRH neurons, since LH secretion was abolished in global Grp54 KO mice, but recovered when Gpr54 expression was selectively reintroduced only in GnRH neurons. It has been shown that kisspeptin neurons in the AVPV express KOR and that these cells are robustly hyperpolarized by a KOR agonist (61). In addition, our results suggest that P does not affect the response of GnRH neurons to kisspeptin as peripheral administration of KP-54 successfully promoted LH secretion in all OVX/E_2_+EB + P-treated mice, consistent with previous research that peripherally administered KP-54 could activate c-FOS in GnRH neurons (37). It therefore appears that the inhibitory effect of P may be mediated by dynorphin and GABA-decreasing kisspeptin release by impairing the activity of the AVPV kisspeptin neurons.

It has been shown that AVPV neurons receive inputs from the suprachiasmatic nucleus (SCN) (63) and that AVPV cells exhibit circadian fluctuations in cAMP levels (64). The time chosen for infusion of drugs in the present study was from 3:00 pm to 7:00 pm on experiment Day 2, which accounts for circadian rhythmicity (35, 36). Our results demonstrate that decreasing GABA_A_ receptor and dynorphin signaling in the AVPV could advance the timing of the LH surge (19, 56), presumably by increasing kisspeptin level to rescue LH surge. These data are supported by previous finding that kisspeptin expression in the AVPV was temporally increased in females in the late afternoon correspondence with circadian time (62, 65).

There is unequivocal evidence that the POA, encompassing the AVPV kisspeptin neurons, is essential for the positive feedback action of E2 that generates the preovulatory GnRH/LH surge in mice and rats (10, 66–68). In the nonrodent species the arcuate KNDy neurons are considered the major population involved in GnRH/LH surge generation (10, 66–68). Although kisspeptin is found in the POA of nonrodent species, including sheep, monkeys, and humans, and kisspeptin expression is elevated at the time of the spontaneous or E2-induced LH surge, the POA is not essential for LH surge generation, at least not in higher primates (66–68). Therefore, there remains the caveat that the mechanisms involved in progesterone-blocking LH surge might be different between rodent and nonrodent species.

In summary, the results of the present study suggest that the progesterone-induced suppression of the LH surge in female mice is mediated by increased dynorphin and GABA_A_ receptor signaling in the AVPV acting though local kisspeptin neurons.

## Additional Information

***Disclosure Summary:*** The authors have nothing to disclose.

***Data Availability.*** All data generated or analyzed during this study are included in this published article or in the data repositories listed in References.
