# ERRATUM FOR “Dynorphin and GABA_A_ Receptor Signaling Contribute to Progesterone’s Inhibition of the LH Surge in Female Mice”

**DOI:** 10.1210/endocr/bqaa071

**Published:** 2020-06-29

In the above-named article by Liu Y, Li X, Ivanova D, Lass G, He W, Chen Q, Yu S, Wang Y, Long H, Wang L, Lyu Q, Kuang Y, and O’Byrne KT (*Endocrinology.* 2020;161(5):1–10; doi: 10.1210/endocr/bqaa036), the following typographical errors in the published paper: author Xi Shen should be listed as third author, between Xiaofeng Li and Deyana Ivanova. This author’s information is as follows:

Xi Shen^1,2,^*

^1^Department of Assisted Reproduction, Shanghai Ninth People’s Hospital Affiliated to Shanghai Jiaotong University School of Medicine, Huangpu District, Shanghai, China; ^2^Department of Women and Children’s Health, Faculty of Life Sciences and Medicine, King’s College London, Guy’s Campus, UK

*Yali Liu, Xiaofeng Li, Xi Shen are joint first authors.

Additionally, author Yanping Kuang should be listed as an additional author for correspondence. This author’s information is as follows:

Yanping Kuang, Department of Assisted Reproduction, Shanghai Ninth People’s Hospital, Shanghai Jiaotong University School of Medicine, Shanghai, People’s Republic of China. E-mail: kuangyanp@126.com.

